# Assessment and Use of Optical Oxygen Sensors as Tools to Assist in Optimal Product Component Selection for the Development of Packs of Ready-to-Eat Mixed Salads and for the Non-Destructive Monitoring of in-Pack Oxygen Levels Using Chilled Storage

**DOI:** 10.3390/foods2020213

**Published:** 2013-05-22

**Authors:** Andreas W. Hempel, Maurice G. O’Sullivan, Dmitri B. Papkovsky, Joseph P. Kerry

**Affiliations:** 1 Food Packaging Group, School of Food and Nutritional Sciences, University College Cork, Cork, Ireland; E-Mails: a.hempel@umail.ucc.ie (A.W.H.); maurice.osullivan@ucc.ie (M.G.O.); 2 Department of Biochemistry, University College Cork, Cork, Ireland; E-Mail: d.papkovsky@ucc.ie

**Keywords:** modified atmosphere packaging, ready-to-eat salads, packaging, storage, sensory, oxygen sensors

## Abstract

Optical oxygen sensors were used to ascertain the level of oxygen consumed by individual salad leaves for optimised packaging of ready-to-eat (RTE) Italian salad mixes during refrigerated storage. Seven commonly found leaves in Italian salad mixes were individually assessed for oxygen utilisation in packs. Each leaf showed varying levels of respiration throughout storage. Using the information obtained, an experimental salad mix was formulated (termed Mix 3) which consisted of the four slowest respiring salad leaves—Escarole, Frisee, Red Batavia, Lollo Rosso. Mix 3 was then compared against two commercially available Italian salads; Mix 1 (Escarole, Frisee, Radicchio, Lollo Rosso) and Mix 2 (Cos, Frisee, Radicchio, Lollo Rosso). Optical sensors were used to non-destructively monitor oxygen usage in all mixes throughout storage. In addition to oxygen consumption, all three salad mixes were quality assessed in terms of microbial load and sensorial acceptability. In conclusion, Mix 3 was found to consume the least amount of oxygen over time, had the lowest microbial load and was most sensorially preferred (*p* < 0.05) in terms of overall appearance and acceptability. This study clearly shows the potential that oxygen sensors possess in terms of assisting in the optimised development of commercial RTE salad products.

## 1. Introduction

The growth in the ready-to-use vegetable market (~10% p.a) has been largely due to increasing demand consumers for fresh, healthy and convenient foods [1]. The most important motivation for purchasing minimally-processed vegetables relates to convenience and speed, especially for consumers who buy these products during their weekend shopping [2]. Consumer demand for freshness and convenience has led to the evolution and increased production of numerous varieties of minimally-processed vegetables presented in a wide range of packaging formats. Vegetables are, in general, highly perishable products that require controlled handling conditions throughout the distribution chain, from producer to consumer, in order to maintain quality and safety and to increase product shelf life [3]. Mixed prepared vegetables deteriorate rapidly and typically possess short shelf lives. Prepared salads comprised of several different components can present unique challenges through widely varying requirements and respiration rates [4]. The shelf-life of ready-to-eat (RTE) vegetable products or salads established by manufacturers is usually 7–14 days depending on the type of fresh produce selected, and is determined by loss in organoleptic qualities [5]. As a result of peeling, grating and shredding, produce will change from a relatively stable product with a shelf-life of several weeks or months to a perishable entity that possesses a very short shelf life, even as short as 1–3 days at chill temperatures. It is possible to achieve a shelf-life of 7–8 days at refrigeration temperatures (5 °C), but for some markets this is not enough and a shelf-life of 2–3 weeks is sometimes necessary [6]. Consequently, modified atmosphere packaging (MAP) technology is largely used for the extended storage of minimally processed fruit and vegetables, including fresh RTE salad products. Oxygen (O_2_), carbon dioxide (CO_2_) and nitrogen (N_2_) are the gases typically implemented in MAP of fresh produce, with O_2_ in packs generally being employed between 1% and 5% in order to support product respiration but used at low enough concentration to discourage the proliferation of microbial spoilage in the form of bacteria and fungi. More recently, the use of high O_2_ atmospheres (*i.e.*, >70% O_2_) have been used as an alternative technique to low O_2_ equilibrium modified atmosphere (3% O_2_) [7]. The concentration of O_2_, as well as CO_2_, within the pack relates to the metabolic state of the produce [1] and indicative of product quality and potential shelf-life.

Optical O_2_ sensors have been shown to provide valuable information in food packaging applications [8,9,10]. The use of a non-destructive method of O_2_ detection to monitor O_2_ levels in different forms of food packaging provides ample information as to the package environment conditions of a food product throughout storage and shelf life. Problems associated with packaging and containment failures can be instantly observed post packaging using this technology [11]. Research has been carried out using O_2_ sensors in specific food applications, such as: MAP cheese [12], vacuum packed cheese [13], MAP and vacuum packed beef [14], cooked meats [15], MAP and vacuum packed chicken [16], as well as *sous vide* products [17]. 

To date, the use of O_2_ sensors have not been employed to monitor O_2_ levels in retail packs of fresh produce and have never been evaluated as a technology to assist in the optimised development of commercial RTE salad products. Therefore, the objective of this study was to employ the use of non-destructive O_2_ sensing technology to ascertain the level of O_2_ consumed by individual salad leaves so that the optimised packaging of RTE Italian salad mixes might be determined. An increase in the O_2_ gas fill of such MAP salads is also used as an alternative to traditional 3%–5% O_2_.

## 2. Experimental Section

### 2.1. Sample Preparation

Salad leaves used in this study were grown in Ireland (and in close proximity to the processing facility where product packaging was undertaken) and consisted of the following salad leaf varieties: Escarole, Frisee, Radicchio, Lollo Rosso, Cos, Iceberg and Red Batavia. Salad leaves undergo treatments such as washing and shredding by manufacturer prior to packaging. Approximately 170 g of each salad leaf was packaged individually for assessment, using an Ishida SE multi head weigher (Ishida Europe, Birmingham, UK) and stored at 4 °C. A Sandiacre Novus 350 (HayssenSandiacre, Duncan, SC, USA) was used to form, fill and seal samples in orientated polypropylene/low density polyethylene laminate films (220 × 290 mm) of 45 micron thickness with modified atmospheres. Control RTE salad mixes were produced commercially with normal packaging conditions (5% O_2_, 5%–10% CO_2_ and 85%–90% N_2_) and monitored for O_2_ levels over time. In order to appropriate the correct level of O_2_ to a mixed salad product, it was necessary to understand the individual O_2_ requirement for each salad leaf within the product mix. Individual salad leaves were packaged using a higher O_2_ level (21% O_2_, 5%–10% CO_2_ and 69%–74% N_2_) to assess the true O_2_ levels utilised by each leaf and subsequent salad mixes during the shelf life period. Mixes of salad leaves were also prepared as described; totalling 170 g, with approximately 25% of each leaf and contents are described in Table 1. Preliminary trials were carried out packaging typical commercial gas (5% O_2_, 5%–10% CO_2_ and 85%–90% N_2_) without salad fill and monitored for oxygen over time. This was undertaken to assess the level of permeation of gases using these commercial packaging materials over a 14 day period. The level of permeation was negligible as sample free packs were seen to hold initial gas fill within <0.5% O2, showing poor permeability.

**Table 1:** Leaf content in salad mixes.

Sample	Leaf content
Mix 1	Escarole, Frisee, Radicchio, Lollo Rosso
Mix 2	Cos, Frisee, Radicchio, Lollo Rosso
Mix 3	Escarole, Frisee, Red Batavia, Lollo Rosso

### 2.2. Optical Sensor Preparation and Readings

Oxygen sensors were prepared using Pt-OEPK (Luxcel Biosciences, Cork, Ireland) spotted on Durapore (Millipore Inc., Bedford, MA, USA) paper using Gilson P100 pipette (Gilson, WI, USA) and cut to a size of 5 mm in diameter. These sensor materials were then placed on 3 cm Avery price marking stickers (Avery Dennison, Pasadena, CA, USA) for adhesion to packaging films. A Mocon OpTech-O_2_ Platinum reader (Mocon Inc., Minneapolis, MN, USA) was used in conjunction with O_2_ sensors allowing for regular non-destructive monitoring of oxygen within packs. The Mocon OpTech-O_2_ device allows for reliable and accurate O_2_ readings from 0.001% to 25% O_2_ in 0.5 s. The device causes excitation of the phosphorescent dye embedded in the optical oxygen sensor and is quenched by molecular oxygen. Calibration of instrumentation was carried out using a Platinum CalCard allowing for instant calibration before each set of measurements were taken. O_2_ readings were taken at day 0, 3, 5, 7 and 10 for commercially prepared MAP RTE salads, individual salad leaves and modified salad mixes. The final measurement was taken on day 10 of storage as no further O_2_ drop was observed and samples had exceeded microbial limits.

### 2.3. Microbial Testing

Microbial testing was carried out during refrigerated shelf-life assessment of individual salad leaves and RTE Italian salad Mixes 1, 2 and 3 on sampling days 1, 3, 5, 7 and 10. Total viable counts (TVC) were carried out to determine microbial numbers (colony forming units (cfu)/g samples) in accordance with ISO standard method (ISO 4833:2003) [18] using total plate count agar and an incubation temperature of 30 °C. The counting of microbial colonies was carried out after 48 h incubation and results were expressed as log values. Results reported up to day seven before exceeding acceptable limits. 

### 2.4. Sensory Analysis

A sensory panel made up of 26 members was recruited in University College Cork, Ireland. Panellists were selected based on their availability on test days and were regular consumers of RTE salad products. All panellists had previous experience in carrying out sensory analysis. A list of descriptors for sensory analysis was selected and these are presented in Table 2. Each panellist was presented with six samples at room temperature (three salad mixes and tested in duplicate) and asked to assess the attributes, according to a 10-point scale. Sensory analysis was carried out during shelf-life evaluation of RTE Italian-style salad products on refrigerated storage days 1, 3 and 7. Sensory analysis was carried out in panel booths conforming to international standards (ISO 8589:2007) [19].

**Table 2:** List of descriptors for sensory analysis.

Attribute-sensory	Descriptor	Scale
*Overall appearance liking*	*The liking of sample appearance*	0 = extremely dislike, 10 = extremely like
*Wilting appearance*	*Presence of leaf wilting*	0 = none, 10 = extreme
*Leaves Superficial Browning (LSB)*	*Appearance of surface leaf browning*	0 = none, 10 = extreme
*Leaves Edge Browning (LEB)*	*Appearance of leaf edge browning*	0 = none, 10 = extreme
*Texture/non-crispy*	*Crispy to non-crispy leaf texture*	0 = none, 10 = extreme
*Off aroma*	*Off aromas*	0 = none, 10 = extreme
*Off flavour*	*Off flavours*	0 = none, 10 = extreme
*Overall flavour liking*	*The overall liking of flavour*	0 = extremely dislike, 10 = extremely like
*Overall acceptability*	*The overall acceptability of the product*	0 = extremely dislike, 10 = extremely like

### 2.5. Statistical Analyses

ANOVA-Partial Least Squares Regression (APLSR) was used to process the mean data accumulated from the 26 panellists during the sensory evaluation. Principal component (PC) 1 *versus* PC 2 is presented; other PC’s did not yield additional information. To derive significance indications for the relationships determined in the quantitative APLSR; regression coefficients were analysed by jack-knifing (Table 3).

**Table 3:** Significance of estimated coefficients (ANOVA *p*-values) for sensory terms as derived by Jack-knife uncertainty testing for Italian-style salad leaf Mixes 1, 2 and 3.

	Salad type			Day storage		
	Mix 1	Mix 2	Mix 3	Day 1	Day 3	Day 7
Overall appearance	−0.38	−0.36	0.005 **	0.007 **	0.47	−0.0003 ***
Wilting appearance	0.41	0.34	−0.009 **	−0.018 *	−0.46	0.0014 **
Leaves Superficial Browning (LSB)	0.53	0.25	−0.21	−0.19	−0.51	0.16
Leaves Edges Browning (LEB)	0.51	0.21	−0.12	−0.14	−0.48	0.093
Texture/non-crispy	0.34	0.41	−0.012 *	−0.016 *	−0.50	0.01 **
Overall flavour	−0.38	−0.47	0.13	0.018*	0.52	−0.03 *
Off aroma	−0.49	−0.64	−0.47	0.51	0.62	−0.51
Off flavour	−0.53	−0.57	−0.44	0.42	0.68	−0.47
Overall acceptability	−0.33	−0.44	0.033 *	0.0046 **	0.51	−0.0041 **

ns = not significant; * = *p* < 0.05; ** = *p* < 0.01; *** = *p* < 0.001.

## 3. Results and Discussion

### 3.1. Oxygen Readings

**Figure 1:**
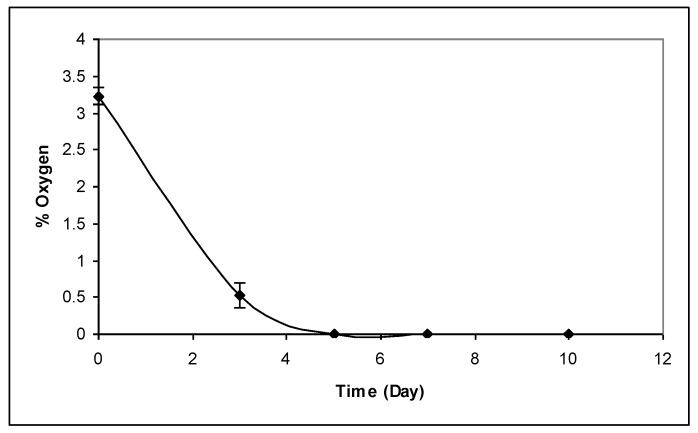
Represents the mean (*n* = 12) oxygen levels in packs of commercially available Italian mix leaves salad (±standard deviation).

In commercial Irish applications, Italian mixed RTE salad leaf products are packaged in low O_2_ modified atmosphere conditions consisting of <5% O_2_. This practice concurs with similar reported practices [20]. In these studies sensors were placed in commercial packs of RTE Italian mixed salads and O_2_ levels were monitored over time. From Figure 1 it is clear that the level of O_2_ provided in such packs may be insufficient for optimised product respiration, as by day 3 of storage, less than 0.5% O_2_ remained within packs. This move towards an anoxic environment leads to quality-linked deteriorative processes within the pack [21,22].

It was necessary to understand the individual O_2_ requirement for each salad leaf within the product mix. Consequently, individual salad leaves typically used within Italian-styled RTE mixed salad leaf products (Escarole, Frisee, Radicchio, Lollo Rosso, Cos, Iceberg and Red Batavia) were packaged individually within MAP formats (21% O_2_, 5%–10% CO_2_ and 69%–74% N_2_) to ensure adequate O_2_ was available for respiration) containing a pre-fitted optical O_2_ sensor. The individual salad leaf packs were monitored over seven days for O_2_ levels to ascertain O_2_ consumption by each salad leaf type. Table 4 shows the amount of O_2_ consumed by each salad leaf type during storage (up to day 7, exceeding microbial limits). The O_2_ differential between leaves showed that after seven days, samples ranged in oxygen utilisation by up 3.2% O_2_. For example, the Radicchio salad leaf was found to consume the greatest amount of O_2_ over the seven days of storage (15.79%), whereas the Lollo Rosso leaf was determined to consume the least amount of O_2_ (12.47%) over the same period of time. The consumption of O_2_ in packs appears to slow after day 7, at which the product is spoilt.

**Table 4:** Consumption of O_2_ by seven individual salad leaf types typically used in Italian-style RTE mixed salad leaf products (including standard deviation).

Salad leaf	Storage (day)					Differential (%)
	Day 0	Day 3	Day 5	Day 7	Day 10	Day 0–7
Escarole	20.82 ± 0.12	13.31 ± 0.32	9.42 ± 0.33	8.20 ± 0.28	8.08 ± 0.17	12.62
Cos	20.63 ± 0.14	10.52 ± 0.51	8.21 ± 1.21	7.34 ± 1.21	7.17 ± 0.92	13.29
Lollo Rosso	20.91 ± 0.11	12.01 ± 0.47	9.80 ± 0.87	8.54 ± 0.82	8.36 ± 0.76	12.37
Frisee	20.52 ± 0.10	12.10 ± 1.58	9.39 ± 2.77	8.01 ± 3.13	7.93 ± 2.14	12.51
Radicchio	20.44 ± 0.08	10.04 ± 0.73	5.78 ± 0.91	4.57 ± 0.90	4.57 ± 0.63	15.87
Iceberg	20.70 ± 0.12	9.09 ± 0.74	6.11 ± 1.12	5.26 ± 1.01	4.88 ± 0.45	15.44
Red Batavia	20.72 ± 0.09	12.31 ± 1.97	8.90 ± 0.84	7.50 ± 0.88	7.29 ± 0.37	13.22

The data generated in this study showed that the amount of O_2_ required by individual salad leaves to respire adequately over a seven day storage period was far greater than the typical <5% O_2_ provided in typical commercial MA packaging applications. This insufficient level of O_2_ utilised in MAP could lead to anaerobic respiration with the production of undesirable metabolites and associated physiological disorders [21]. 

It is apparent that each leaf respires at different rates under the same conditions. The level of O_2_ utilisation can be categorised in a table representing each salad leaf in the order of increasing respiratory levels. Table 5 outlines O_2_ consumption by individual salad leaves in order of increasing O_2_ utilisation on storage days 3, 5 and 7. Escarole, Lollo Rosso and Frisee were consistently the slowest respiring salad leaves while Cos, Radicchio and Iceberg were consistently found to utilise the greatest amount of in-pack O_2_. Individually packed leaves underwent microbial (TVC) testing.

**Table 5:** Ranking of individual salad leaf types in terms of O_2_ consumption within refrigerated MA packs; lowest O_2_ consumption (1) to highest O_2_ consumption (7).

Ranking	Day 3	Day 5	Day 7	Day 10
1	Escarole	Lollo Rosso	Lollo Rosso	Lollo Rosso
2	Red Batavia	Escarole	Escarole	Escarole
3	Frisee	Frisee	Frisee	Frisee
4	Lollo Rosso	Red Batavia	Red Batavia	Red Batavia
5	Cos	Cos	Cos	Cos
6	Radicchio	Iceberg	Iceberg	Iceberg
7	Iceberg	Radicchio	Radicchio	Radicchio

### 3.2. Microbial Testing

Each individually packed salad leaf was measured by total viable count (TVC) assay. Testing was carried out on day 1, 3, 5, 7 and 10 to establish the microbial load in colony forming units per gram of sample. Table 6 represents the log cfu/g of each salad leaf on each testing day. With the exception of Cos and Iceberg, all other total viable counts appear to be quite similar between log 5 and log 6 by day 5. With the exception of Iceberg, all salads were deemed unfit for consumption by day 7 as all counts had exceeded log 6. Cos had the lowest counts on day 1 of storage, but by day 7 were found to be quite similar to all other leaves. Iceberg was the only leaf that had lower microbial numbers than the rest, with its final count being log 6.1 at day 10, exceeding acceptable limits. Microbial counts determined in this study over time concur with that reported in the scientific literature [22,23]. 

**Table 6:** Table represents the mean log cfu/g of each salad leaf (±standard deviation).

Salad leaf	Storage (day)				
	Day 1	Day 3	Day 5	Day 7	Day 10
Cos	2.2 ± 0.27	3.1 ± 0.47	4.3 ± 0.16	6.3 ± 0.09 ^	n/d
Escarole	4.5 ± 0.14	4.9 ± 0.09	5.3 ± 0.28	6.5 ± 0.04 ^	n/d
Frisee	4.8 ± 0.09	5.1 ± 0.17	5.5 ± 0.19	6.5 ± 0.08 ^	n/d
Lollo Rosso	4.3 ± 0.22	4.8 ± 0.31	5.4 ± 0.14	6.0 ± 0.13 ^	n/d
Radicchio	4.6 ± 0.34	5.0 ± 0.07	5.8 ± 0.21	6.5 ± 0.12 ^	n/d
Red Batavia	4.5 ± 0.29	5.1 ± 0.19	5.6 ± 0.17	6.5 ± 0.09 ^	n/d
Iceberg	3.0 ± 0.08	3.8 ± 0.70	4.3 ± 0.05	5.6 ± 0.04 ^	6.1 ± 0.07 ^

^ Exceeds limits of 10^6^ or 6 log cfu/g; n/d—not determined.

In the case of Radicchio, a correlation between highest level of oxygen utilisation of all leafs and microbial counts at day 7 are observed. The inverse of this can be seen with Lollo Rosso, where it was found to consume the lowest level of O_2_ over 10 days and had the second lowest log TVC value. Results can also be ranked in terms of which salad leaf has the lowest microbial load in order to understand the difference in microbial quality across all samples. This can be seen in Table 7, where salad leaves are listed in order of lowest microbial counts (1) (log cfu/g sample) to highest (10). From this information it can be seen that at day 1, Cos and Frisee are the lowest and highest microbial load, respectively. By day 7 Iceberg and Radicchio has the lowest and highest microbial load, respectively. 

**Table 7:** Salad leaves ranked in terms of lowest log cfu/g (1 to 7) on day 1, 3, 5 and 7

Rank	Day 1	Day 3	Day 5	Day 7
1	Cos	Cos	Cos	Iceberg
2	Iceberg	Iceberg	Iceberg	Lollo Rosso
3	Lollo Rosso	Escarole	Escarole	Cos
4	Red Batavia	Lollo Rosso	Lollo Rosso	Red Batavia
5	Escarole	Frisee	Frisee	Frisee
6	Radicchio	Red Batavia	Red Batavia	Escarole
7	Frisee	Radicchio	Radicchio	Radicchio

### 3.3. Shelf-Life Comparison of an Experimentally Formulated Map Italian Salad Leaf Mix with Two Commercial Forms

Based on the results obtained by monitoring the level of O_2_ utilised by each salad leaf (from seven leaf types typically used to construct Italian style mixed leaf RTE salads), an experimental salad mix was formulated using the four lowest respiring leaves determined by the O_2_ sensor previously. This Italian-styled formulation (Mix 3) and two similar commercially available formulations (Mixes 1 and 2) were modified atmosphere packaged using the same packaging conditions, in the same manufacturing plant, using salad materials grown and processed in the same manner. All three salad mixes were produced in volume and a shelf-life evaluation study undertaken. Oxygen measurements were taken throughout product storage and optical O_2_ sensor readings showed that Italian salad Mix 3 had the lowest O_2_ consumption of all three salad mixes (Table 8). After seven days of storage, Salad Mix 3 had a residual O_2_ level of 9.25% remaining in packs compared to 8.12% in Mix 1 and 7.61% in Mix 2. As outlined previously, salad Mix 3 was formulated using the four slowest respiring leaves and so it makes logical sense that packs containing this product mix had the highest remaining O_2_ found in packs over the four days of sampling. The concentration of oxygen inside the headspace is related to the metabolic state of the samples [1]. Commercial salad Mixes 1 and 2 both use the Radicchio leaf which was found to be the highest O_2_ consumer (15.79%) of all seven leaf types assessed previously and commonly found in Italian-style mix leaf salads. Salad Mix 2 was also comprised of the Cos leaf, which was also found to be amongst the highest O_2_ consumers (13.33%) of all leaves assessed. Consequently, the use of Radicchio and other high respiring leaves in mixes ultimately results in a final product that consumes greater amounts of O_2_. Therefore, it was essential to provide a modified atmosphere with enough O_2_ to allow sufficient O_2_ for respiration irrespective of what leaves selected. It is important to recognize that extremely low O_2_ levels or excessively high CO_2_ levels can result in the generation of off-flavours or visible tissue damage [24]. 

**Table 8:** Represents the mean % O_2_ by optical sensor (*) and log cfu/g incl. standard deviation (^) found in each mix on day 0, 3, 5 and 7.

Sample	Leaves	Day storage				
		Day 0	Day 3	Day 5	Day 7	Day 10
Mix 1 *	Escarole, Frisee, Radicchio, Lollo Rosso	20.33 ± 0.11	13.45 ± 1.54	13.45 ± 1.54	8.13 ± 1.45	7.93 ± 1.22
Mix 2 *	Cos, Frisee, Radicchio, Lollo Rosso	20.18 ± 0.10	12.98 ± 1.19	12.98 ± 1.19	7.61 ± 1.80	7.11 ± 1.46
Mix 3 *	Escarole, Frisee, Red Batavia, Lollo Rosso	20.64 ± 0.05	14.69 ± 0.59	14.69 ± 0.59	9.25 ± 0.59	8.15 ± 0.37
Mix 1 ^	Escarole, Frisee, Radicchio, Lollo Rosso	3.24 ± 0.34	4.84 ± 0.24	5.52 ± 0.30	6.81 ± 0.28	n/d
Mix 2 ^	Cos, Frisee, Radicchio, Lollo Rosso	3.12 ± 0.15	4.00 ± 0.21	5.31 ± 0.23	6.21 ± 0.31	n/d
Mix 3 ^	Escarole, Frisee, Red Batavia, Lollo Rosso	2.67 ± 0.17	3.86 ± 0.12	4.97 ± 0.09	5.79 ± 0.20	6.21 ± 0.27

n/d—not determined.

All three salad leaf mixes were assessed for microbial quality during refrigerated storage (Table 8). Microbial counts for salad Mix 1 and Mix 2 were found to yield similar results on all days of analysis throughout storage and have exceeded acceptable limits by day 7. Results showed that salad Mix 3 consistently had lower microbial counts on each sampling day throughout storage when compared to commercial salad Mixes 1 and 2. It was clear that the initial microbial counts at day 0 were much lower in Mix 3, where the selection of slower respiring leaves had resulted in a product requiring less O_2_ over time. By day 7, Mix 3 was found to still maintain acceptable limits of <log 6 [22,23], where log 5.79 was recorded. The use of salad leaf mixes which are found to use less O_2_ over time appears to result in a product that has a lower microbial count than that of one which consumes more.

### 3.4. Sensory and Statistical Analysis

**Figure 2:**
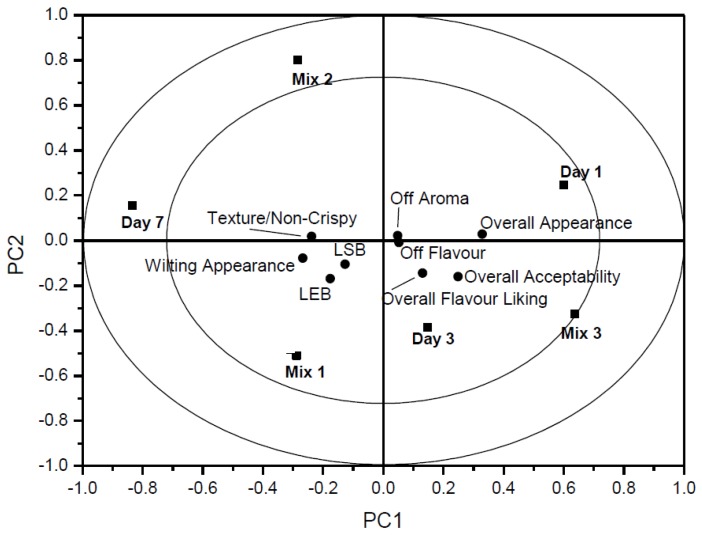
An overview of the variation found in the mean data from the ANOVA-partial least squares regression (APLSR) correlation loadings plot for each of the three salad mixes.

Salad Mixes 1, 2 and 3 were assessed for sensory acceptability over the seven day storage period (days 1, 3 and 7). Panellists were made aware of relevant descriptors (Table 2) and rating scale. The shelf-life of all salad mixes was perceived in a predictable manner, where day 1 showed all salads to be in a “Fresh” state, where overall appearance (*p* ≤ 0.01), flavour (*p* ≤ 0.05) and acceptability (*p* ≤ 0.01) were significantly and positively correlated. Day 7 data contrasted significantly with salad samples found to be negatively correlated with overall appearance, flavour and acceptability, along with a positive correlation for wilting appearance (*p* ≤ 0.01) and non-crispy texture (*p* ≤ 0.01). As for the individual salads, it was apparent that salad Mix 3 was favoured amongst panellists over salad Mixes 1 and 2, where overall appearance and acceptability were significantly correlated (*p* ≤ 0.01) and descriptors such as wilting appearance and non-crispy texture were found to be negatively correlated (*p* ≤ 0.05) showing a true preference for salad Mix 3. This selection appears to be attained by the use of Red Batavia instead of Radicchio. Panellists were asked if any individual leaf was found to be particularly undesirable and in this case 16 of the 26 panellists (61.5%) found the Radicchio leaf to be undesirable within the salad mix. The Radicchio leaf is understood to be a spicier and a more bitter intense leaf [25] which most panellists found undesirable when compared to the Red Batavia alternative. Both salad Mixes 1 and 2 had no significantly positive or negative attributes associated when compared with each other. The use of higher levels of O_2_ (21%) in packaging did not appear to have an adverse affect on browning of the salad leaves. Data derived by Jack-Knifing uncertainty testing for individual salad leaves find no significance in LSB or LEB. Figure 2 represents an overview plot of the mean data from the ANOVA correlation values for all three mixes.

With the understanding that Mix 3 was found to be the most desirable in terms of sensory analysis, the formulation of a mix with a lower microbial load and slower respiration rate compared well to common commercially produced salad mixes. The use of an optical O_2_ sensor in determining the oxygen respiration levels shows a novel and effective tool in understanding the O_2_ levels used in respiring salads leaves packaged in modified atmosphere conditions. This valuable information let the development of a product with a longer, more stable shelf life and a product more acceptable to consumers.

## 4. Conclusions

The ability to non-destructively assess the level of O_2_ utilised by individual salad leaves was successfully achieved using optical O_2_ sensors. Information gathered using O_2_ sensors showed that an O_2_ level in excess of 10% is required to provide salad leaves with enough O_2_ to comfortably respire within a typical shelf-life period of seven days. The apparent lack of O_2_ within such packs has been previously shown to cause many deleterious quality processes. The use of optical O_2_ sensors showed that a salad mix can be optimised by understanding specific product requirements, like that for oxygen, and subsequently, careful product selection; thereby assisting in extending product shelf-life and promoting quality.

## References

[1] Rico D., Martin-Diana A.B., Barat J.M., Barry-Ryan C. (2007). Extending and measuring the quality of fresh cut fruit and vegetables: A review. Trends Food Sci. Technol..

[2] Ragaert P., Verbeke W., Devlieghere F., Debevere J. (2003). Consumer perception and choice of minimally processed vegetables and packaged fruits. Food Qual. Prefer..

[3] Pocas M.F.F., Delgado T.F., Oliveira F.A.R. (2008). Smart Packaging Technologies: Smart Packaging Technologies for Fruits and Vegetables.

[4] Sandhya (2010). Modified atmosphere packaging of fresh produce: Current status and future needs. LWT Food Sci. Technol..

[5] Garcia-Ginemo R.M., Zurera-Cosano G. (1997). Determination of ready to eat vegetable salad shelf life. Int. J. Food Microbiol..

[6] Ahvenainen R. (1997). New approaches in improving the shelf life of minimally processed fruit and vegetables. Trends Food Sci. Technol..

[7] Jacxsens L., Devlieghere F., van der Steen C., Debevere J. (2001). Effect of high oxygen modified atmosphere on microbial growth and sensorial qualities of fresh cut produce. Int. J. Food Microbiol..

[8] Papkovsky D.B. (1995). New oxygensensors and their application to bio sensing. Sens. Actuators B Chem..

[9] Papkovsky D.B. (2004). Methods in optical oxygen sensing: Protocols and critical analyses. Methods Enzymol..

[10] O’Riordan T.C., Voraberger H., Kerry J.P., Papkovsky D.B. (2005). Study of migration of active components of phosphorescent oxygen sensors for food packaging applications. Anal. Chim. Acta.

[11] Hempel A.W., O’Sullivan M.G., Papkovsky D.B., Kerry J.P. (2013). Use of optical oxygen sensors to monitor residual oxygen in pre- and post-pasteurised bottled beer and its affect on sensory attributes and product acceptability during simulated commercial storage. LWT Food Sci. Technol..

[12] O’Mahoney F., O’Riordan T.C., Papkovskaia N., Kerry J.P., Papkovsky D.B. (2006). Non-destructive assessment of oxygen levels in industrial modified atmosphere packaged cheddar cheese. Food Control.

[13] Hempel A.W., Gillanders R.N., Papkovsky D.B., Kerry J.P. (2012). Detection of cheese packaging containment failures using reversible optical oxygen sensors. Int. J. Dairy Technol..

[14] Smiddy M., Fizgerald M., Kerry J.P., Papkovsky D.B., O’Sullivan C.K., Guilbault G.G. (2002). Use of oxygen sensors to non-destructively measure the oxygen content in modified atmosphere and vacuum packed beef: Impact of oxygen content on lipid oxidation. Meat Sci..

[15] Smiddy M., Papkovsky D.B., Kerry J.P. (2002). Evaluation of oxygen content in commercial modified packs (MAP) of processed cooked meats. Food Res. Int..

[16] Smiddy M., Papkovskaia N., Papkovsky D.B., Kerry J.P. (2002). Use of oxygen sensors for the non-destructive measurement of the oxygen content in modified atmosphere and vacuum packs of cooked chicken patties; impact of oxygen content on lipid oxidation. Food Res. Int..

[17] O’Mahoney F., O’Riordan T.C., Papkovskaia N., Ogurtsov V.I., Kerry J.P., Papkovsky D.B. (2004). Assessment of oxygen levels in convenience-style muscle based *sous vide* products through optical means and impact on shelf life stability. Pack. Technol. Sci..

[18] ISO (the International Organization for Standardization) (2003). Microbiology of Food and Animal Feeding Stuffs—Horizontal Method for the Enumeration of Microorganisms—Colony-Count Technique at 30 Degrees C, ISO 4833:2003.

[19] ISO (the International Organization for Standardization) (2007). Sensory Analysis—General Guidance for the Design of Test Rooms, ISO 8589:2007.

[20] Rojas-Grau M.A., Oms-Oliu G., Soliva-Fortuny R., Martin-Belloso O. (2009). The use of packaging techniques to maintain freshness in fresh-cut fruits and vegetables: A review. Int. J. Food Sci. Technol..

[21] Soliva-Fortuny R.C., Martin-Belloso O. (2003). New advances in extending the shelf life of fresh-cut fruits: A review. Trends Food Sci. Technol..

[22] Harrigan W.F. (1998). Laboratory Methods in Food Microbiology; Microbiological Examinations in Vegetables.

[23] Szabo E.A., Scurrah K.J., Burrows J.M. (2002). Survey for psychrotropic bacterial pathogens in minimally processed lettuce. Lett. Appl. Microbiol..

[24] Beaudry R.M. (1998). Effect of O_2_ and CO_2_ partial pressure on selected phenomena affecting fruit and vegetable quality. Postharvest Biol. Technol..

[25] Tordoff M.G., Sandell M.A. (2009). Vegetable bitterness is related to calcium content. Appetite.

